# A Literature Review of the Incidence, Management, and Prognosis of Corneal Epithelial-Related Complications After Laser-Assisted In Situ Keratomileusis (LASIK), Photorefractive Keratectomy (PRK), and Small Incision Lenticule Extraction (SMILE)

**DOI:** 10.7759/cureus.43926

**Published:** 2023-08-22

**Authors:** Majid Moshirfar, Jordan M Santos, Qiancheng Wang, Isabella M Stoakes, Kaiden B Porter, Josh S Theis, Phillip C Hoopes

**Affiliations:** 1 Corneal and Refractive Surgery, Hoopes Vision Research Center, Draper, USA; 2 Ophthalmology and Visual Sciences, John A. Moran Eye Center, University of Utah, Salt Lake City, USA; 3 Eye Banking and Corneal Transplantation, Utah Lions Eye Bank, Murray, USA; 4 Medicine, University of Arizona College of Medicine, Phoenix, USA; 5 Medicine, Baylor College of Medicine, Houston, USA; 6 Osteopathic Medicine, Pacific Northwest University of Health Sciences, Yakima, USA; 7 Ophthalmology, Hoopes Vision Research Center, Draper, USA

**Keywords:** epithelial sloughing, microstriae, epithelial ingrowth, lamellar keratitis, recurrent corneal erosions, smile, prk, lasik

## Abstract

Our purpose is to provide a comprehensive investigation into the incidence, treatment modalities, and visual prognosis of epithelial-related complications in corneal refractive surgeries, including laser-assisted in situ keratomileusis (LASIK), photorefractive keratectomy (PRK), and small incision lenticule extraction (SMILE). A systematic search of multiple databases was conducted by two independent examiners using various search terms related to epithelial-related complications and corneal refractive surgeries. A total of 91 research articles were included, encompassing a sample size of 66,751 eyes across the three types of surgeries. The average incidence of epithelial-related complications varied across the different types of corneal refractive surgeries. LASIK had an average incidence of 4.9% for epithelial defects, while PRK and SMILE had lower rates of 3.3% and 3.9%, respectively. Our findings indicate that SMILE has a lower incidence of epithelial defects compared to LASIK, potentially due to the less invasive nature of lenticule incision in SMILE. Visual prognosis after epithelial complications (EC) is generally favorable, with various supportive care and surgical interventions leading to significant improvements in postoperative visual acuity and full recovery. Understanding the incidence rates and management approaches for epithelial-related complications can guide clinicians in enhancing patient safety, refining surgical techniques, and optimizing postoperative outcomes in corneal refractive surgeries.

## Introduction and background

It is estimated that 800,000 to 1.4 million corneal refractive surgeries are performed annually in the United States, including procedures such as photorefractive keratectomy (PRK), laser in-situ keratomileusis (LASIK), and small incision lenticule extraction (SMILE) [1,2]. However, both intraoperative and postoperative epithelial-related complications are known to be associated with corneal refractive surgeries. Common complications include epithelial ingrowths (EIs), microstriae, filamentary keratitis (FK), diffuse lamellar keratitis (DLK) secondary to epitheliopathy, recurrent corneal erosions (RCEs), and epithelial defects such as abrasions.

These complications arise as a result of abnormal corneal epithelial healing, a complex process involving various cytokines, neurotrophic growth factors, and cell types. Immediately following insult, cytokines are activated and induce a response in keratocytes. These keratocytes then release growth factors and interleukins, which regulate the proliferation and migration of keratocytes, facilitating the transformation of keratocytes into myofibroblasts. If the corneal stroma is injured, myofibroblasts produce glycosaminoglycans and extracellular matrix components to facilitate its reconstruction. In healthy individuals, this healing process can typically span from a few days to as long as eight weeks, particularly if the basement membrane is disrupted [1,3,4].

The incidence of epithelial-related complications associated with corneal refractive surgeries varies depending on the specific procedure. Nevertheless, these complications may share similar underlying pathophysiology and risk factors, which can increase the likelihood of patients developing long-term epithelial complications (EC) postoperatively. This article aims to provide a thorough review of both intraoperative and postoperative epithelial-related complications associated with corneal refractive surgeries, including the identification of risk factors, the incidence of complications, management strategies, and respective prognoses.

## Review

Methods

**Figure 1:**
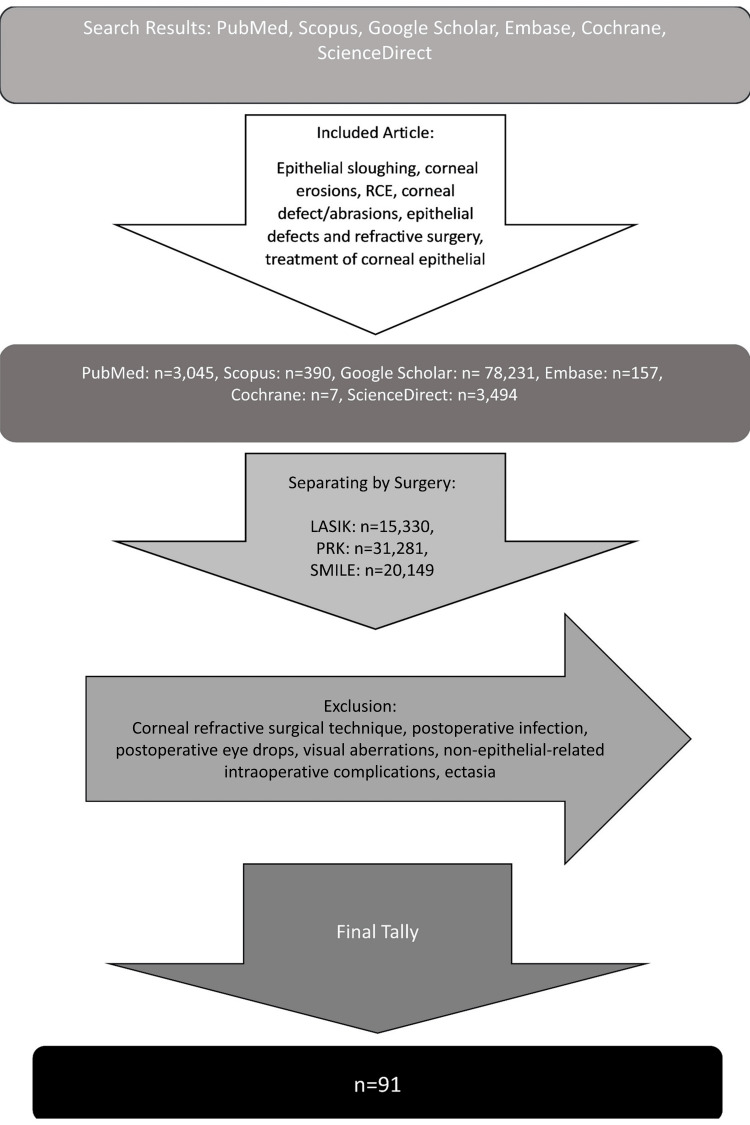
Literature Search Method RCE, recurrent corneal erosion; PRK, photorefractive keratectomy; SMILE, small incision lenticule extraction; LASIK, laser-assisted in situ keratomileusis

Two independent examiners utilized PubMed, Google Scholar, Scopus, Embase, Cochrane, and ScienceDirect to find articles. The following search terms were used: “LASIK, epithelial sloughing,” “LASIK, epithelial defect,” “LASIK, corneal erosions,” “PRK, epithelial sloughing,” “PRK, recurrent corneal erosions,” “SMILE, corneal erosions,” “SMILE, corneal abrasions,” “SMILE epithelial defect,” “small incision lenticule extraction, corneal defects,” “recurrent corneal erosions,” “epithelial defect and refractive surgery,” “anterior basement membrane dystrophy,” “SMILE diffuse lamellar keratitis,” “LASIK diffuse lamellar keratitis,” “PRK diffuse lamellar keratitis,” “SMILE filamentary keratitis,” “LASIK filamentary keratitis,” “PRK filamentary keratitis,” and “Treatment of epithelial defects.” Articles were classified according to the type of corneal refractive surgery with n=31,281 for PRK, n=15,330 for LASIK, and n=20,140 for SMILE. Within each type of surgery, further articles were subdivided into groups detailing epithelial sloughing, recurrent corneal erosions, corneal defect, epithelial defect and refractive surgery, corneal abrasions, diffuse lamellar keratitis, filamentary keratitis, and treatment of corneal epithelial defects. Journal articles, literature reviews, case reports, and case series were included, while brief reports and books were excluded from this search. Additionally, journal articles and case reports focusing on surgical technique, postoperative infection, dry eyes, visual aberrations, non-epithelial-related intraoperative complications, and ectasia were excluded. Research articles before 1990 were excluded. A total of 91 research articles were identified and utilized in this literature review (Figure 1).

The average incidence of epithelial-related complications across multiple studies of LASIK, PRK, and SMILE was calculated. The incidence ranges of epithelial complications were reported directly from the literature or reporting the lowest and highest incidence for each defect. We defined epithelial-related complications (intraoperative and postoperative) as the following: FK, DLK, microstriae secondary to epithelial sloughing, RCE, EI, and epithelial defects such as abrasions during acute phase of surgery. The following were excluded from the definition of epithelial complications (EC): corneal staining, conjunctival staining, punctate epithelial keratopathy, superficial punctate keratitis, and scaling of dry eyes. The treatment modalities of epithelial-related complications and visual prognosis were also noted and included in this literature review.

It is important to note that incidence can be defined as the number of new cases of a condition during a specific time interval as indicated in biostatistics [5]. However, in the calculation of incidence or in reporting the range of incidence for these studies, averages were calculated across multiple retrospective studies that reported the rates of epithelial-related complications as incidence. These studies spanned different time periods.

Results

**Figure 2:**
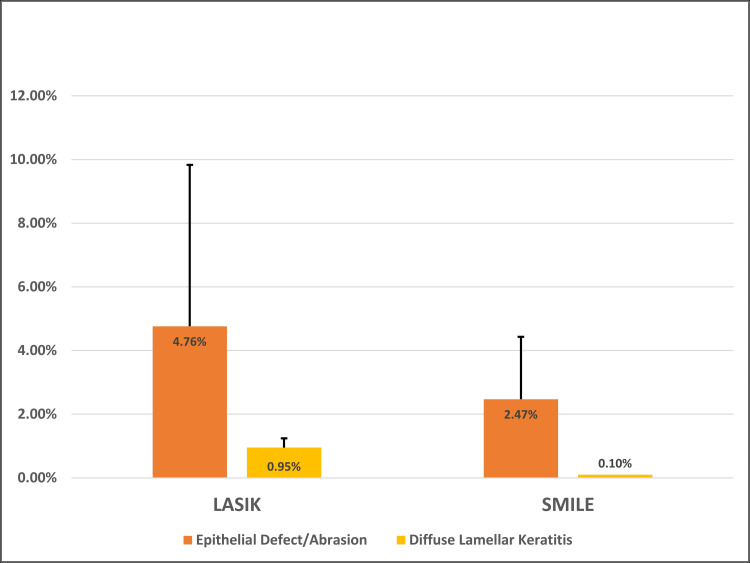
Average Incidence of Epithelial Complications Across LASIK and SMILE LASIK, laser in-situ keratomileusis; SMILE, small incision lenticule extraction

**Figure 3:**
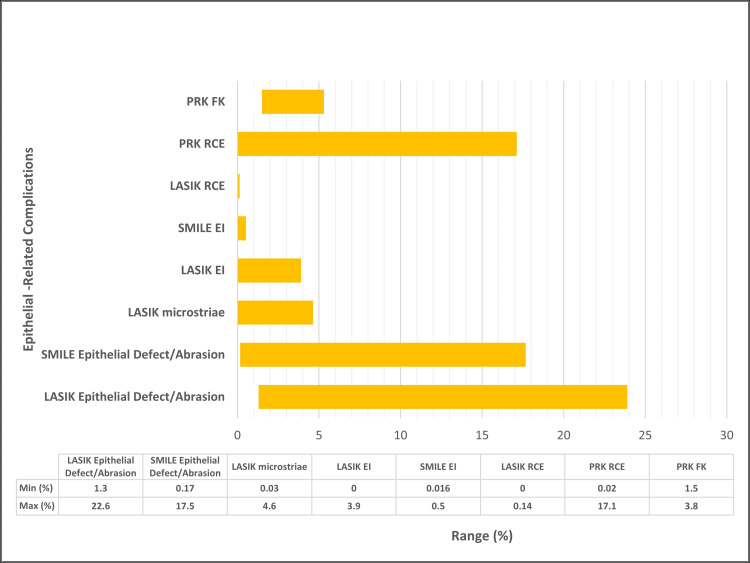
Incidence Range of Epithelial-Related Complications Across LASIK, SMILE, and PRK per Literature LASIK, laser in-situ keratomileusis; PRK, phototherapeutic keratectomy; SMILE, small incision lenticule extraction; EI, epithelial ingrowth; RCE, recurrent corneal erosion; FK, filamentary keratitis; min, minimum; max, maximum

The following epithelial-related complications will be elaborated with the corresponding corneal refractive surgery (LASIK, PRK, and SMILE): epithelial defects/abrasions (Figure 2), RCE, DLK secondary to epitheliopathy, EI, microstriae, and FK (Figure 3).

Average incidence for epithelial defects/abrasions and DLK associated with LASIK and PRK was calculated. For the remaining categories of epithelial-related complications, incidence ranges were reported.

Intraoperative epithelial defect/abrasions

LASIK

**Figure 4:**
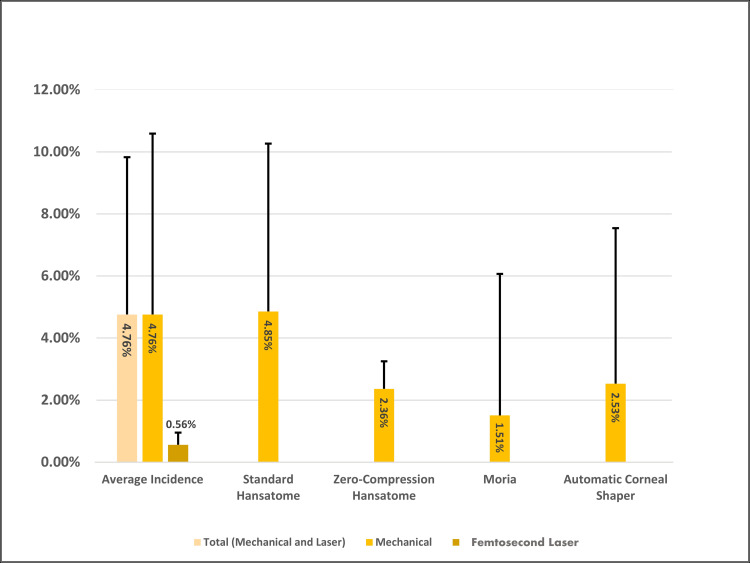
Incidence of Epithelial Defects Across Devices for LASIK Flap Creation LASIK: laser in-situ keratomileusis

During the creation of the LASIK flap, epithelial or corneal abrasions frequently emerge at the flap edges [6-8]. The average incidence of epithelial defects or abrasions, calculated from 15 studies on LASIK, was found to be 4.9%, with a range of incidence from 1.3% to 22.6% (Figure 4).

The risk factors for epithelial defects were also identified. Tekwani and Huang reported age as a risk factor, with an odds ratio (OR) increase of 2.39 per decade of life. The use of topical anesthetics was another identified risk factor. The OR for developing epithelial abrasions increased in groups treated with additional topical proparacaine instead of lubricating eye drops. Corneal thickness also played a role as a risk factor, with an OR increase of 2.3 per 50 µm increase in thickness [9]. Bashour reported that lighter skin tone and hair color, along with blue eyes, were risk factors for intraoperative epithelial abrasions [10]. One of the most significant risk factors was a history of epithelial defects during LASIK [11,12]. When one eye developed an intraoperative abrasion, the conditional probability of a defect occurring in the second eye increased to 66.7% [13].

The choice of instruments used for LASIK flap creation can also impact the incidence of epithelial defects [14-16]. Mechanical instruments, such as the Hansatome microkeratome, utilize a circular cutting blade for flap creation. Within the Hansatome category, there is the standard and the zero-compression series, which uses a flatter head. In the standard Hansatome series, epithelial defect incidence is reported between 1.6% and 14.5% [9,12,17,18]. A study comparing the standard Hansatome to the zero-compression series found that the incidence of epithelial defects was 8.8% versus 2.7%, respectively [19]. In a study performed by Kohnen et al. using inter-eye comparison where one eye underwent standard keratome while the other zero compression for flap creation, the incidence of epithelial defect was 22.6% in the standard group compared to 2.1% in the zero-compression group [20]. Another less widely used mechanical instrument is the automatic corneal shaper (ACS), which utilizes a linear blade for flap creation. The occurrence of epithelial defects in this design is lower, perhaps from the lack of torsional stress placed on the epithelium during flap cut. In a study of 1,873 eyes, Chen et al. found an incidence of 1.66% using the ACS [13].

Femtosecond LASIK (FS-LASIK) serves as an alternative to mechanical LASIK (m-LASIK), as it employs a femtosecond laser for flap creation, thereby eliminating the stress forces generated by a microkeratome. In a direct comparison study of 1,798 eyes by Moshirfar et al., it was found that 2.6% of patients in the zero-compression cohort developed defects larger than 4 mm², compared to 0.6% in the FS-LASIK cohort [21]. Two meta-analyses, comprising 11 randomized control studies and five cohort studies, reached a similar conclusion, suggesting that FS-LASIK has a lower incidence of epithelial defects compared to mechanical LASIK (m-LASIK) (Table 1) [13,22].

**Table 1:** Summary of Studies Reporting the Incidence of Epithelial Defect Associated With LASIK LASIK: laser-assisted in situ keratomileusis

Study	Instrument	Eyes (patients)	Percentage of the incidence of epithelial defect (eyes)
Kohnen et al. (2016) [1]	Standard	186 (93)	22.6% (21)
	Zero-compression Hansatome		2.1% (two)
Kenyon et al. (2004) [2]	Moria LSK-One	500 (286)	10.20% (51)
Tekwani and Huang (2002) [9]	Hansatome	247 (133)	9.70% (24)
Bashour (2002) [10]	Hansatome	1,852 (926)	14% (259)
Mirshahi et al. (2004) [11]	Technolas C-LASIK 217 and Hansatome microkeratome	1650	1.3% (22)
Smirennaia et al. (2001) [12]	Nidek EC-5000 and Chiron Vision HT-280 microkeratome	5,896 (2,972)	1.60% (95)
Chen et al. (2007) [13]	ACS (Chiron)	1,873 (956)	1.66% (31)
Kezirian and Stonecipher (2004) [14]	Femtosecond laser	106	0%
	Moria	126	9.6%
	Hansatome	143	7.7% (P=0.001)
Randleman et al. (2007) [15]	Nidek EC-5000	6,984 (6,067 myopes and 917 hyperopes)	647 (487 myopes {8%} and 160 hyperopes {17.3%})
	LADARVision laser		
Ahee et al. (2002) [16]	Nidek EC-5000 and Moria C-B microkeratome	BSS, 96 (48); Refresh Plus, 114 (57)	BSS: 13.5% (13)
			Refresh Plus: 3.5% (4) (P=0.02)
Jabbur and O’Brien (2003) [17]	Hansatome versus Amadeus (ACS)	263 (133)	7.2% (19) versus 1.5% (four)
Polk et al. (2005) [19]	Standard	216	8.8% (19)
	Zero-compression Hansatome	188	2.7% (five)
Moshirfar et al. (2010) [21]	Zero-compression microkeratome	896 (493)	2.6% (23)
	Femtosecond laser		0.6% (5) (P=0.0006)
Pérez-Santonja et al. (2005) [23]	K-E (Chiron)	5,670 LASIK procedures	0.32% (18)
	BD K-3000 (Becton Dickinson)		
	M2 (Moria) microkeratome		

SMILE

**Figure 5:**
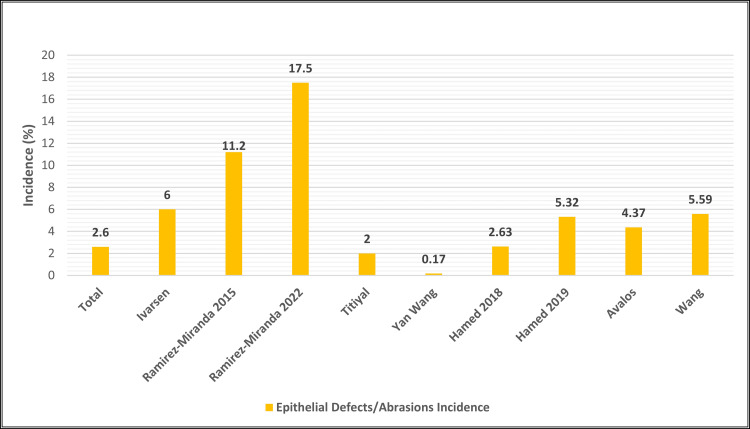
Incidence of Epithelial Defects/Abrasions Across SMILE Studies SMILE: small incision lenticule [24-30,37,38]

Epithelial defects occur less frequently in SMILE compared to LASIK [24-27]. Visual prognosis following treatment is generally favorable [24-38]. The average incidence of epithelial defects in SMILE is 3.3% with a reported incidence range of 0.17%-17.5%, based on nine studies (Figure 5 and Table 2) [24-30,37,38].

**Table 2:** Summary of Studies Reporting the Incidence of Epithelial Defect Associated With SMILE SMILE, small incision lenticule extraction; NP, not provided

Study	Instrument	Eyes (patients)	Percentage of the incidence of corneal epithelial defects (eyes)
Hamed et al. (2019) [24]	VisuMax FS (Carl Zeiss Meditec)	282 (141)	5.32% (15)
Ramirez-Miranda et al. (2022) [25]	NP	228	17.5% (40)
Ivarsen et al. (2014) [26]	VisuMax FS (Carl Zeiss Meditec)	1,574 (922)	6% (94)
Avalos et al. (2019) [27]	VisuMax platform	526 (263)	Intraoperative: 2.85%
			Postoperative: 1.52%
Ramirez-Miranda et al. (2015) [28]	NP	160	11.2% (18)
Wang et al. (2017) [29]	VisuMax FS (Carl Zeiss Meditec)	6,373 (3,223)	0.14% striae
			0.02% ingrowth
Titiyal et al. (2017) [30]	VisuMax FS (Carl Zeiss Meditec)	100 (50)	2% (two)
Hamed et al. (2018) [37]	VisuMax FS (Carl Zeiss Meditec)	190	2.63% (five)
Wang et al. (2019) [38]	VisuMax FS (Carl Zeiss Meditec)	3,004 (1,511)	0.17% (five)

One study involving 3,004 eyes reported a 0.17% incidence of abrasion with the use of a bandage contact lens (BCL), and all cases achieved uncorrected distance visual acuity (UDVA) of 20/20 or better at six months postoperatively [29]. Abrasions typically occur around the incision site, while central erosions have rarely been reported. For instance, Titiyal et al. noted an incidence of 0.3% in their study of 1,800 eyes [30].

Ramirez-Miranda et al. evaluated both surgical outcomes and complications of SMILE with fellows under attending supervision finding that epithelial defects were the most common postoperative complication accounting for 17.5% incidence in a data set of 228 eyes. The authors suggested that the higher rate of epithelial defects could be attributed to the limited experience of the corneal fellows, potentially resulting in challenges in identifying anterior or posterior lenticular planes and less attention being given to the integrity of the incision site. Also, Ramirez-Miranda et al.’s study surveilled 160 eyes undergoing SMILE for postoperative complications and found that the most common postoperative complication was epithelial defect, representing 11.2% incidence. Once again, the authors attributed this to surgical experience as these defects occurred with surgeons that were acclimating to the technique required to dissect and extract a lenticule, noting that after 4-6 SMILE surgeries, the defect disappeared [25].

The treatment for intraoperative epithelial erosion is generally uncomplicated (Table 3).

**Table 3:** Summary of Corneal Epithelial Defect Treatments N/A, not applicable; BCL, bandage contact lens

Treatment type	First line	Second line	Novel
Medical	Treat underlying condition (diabetes and Sjögren’s syndrome) or remove iatrogenic cause	Autologous serum drops	Topical fibronectin, beta 4, or fibronectin-derived peptide
	Artificial tears/ocular ointment	Umbilical cord blood serum or platelet-rich fibrin drops	Lufepirsen ophthalmic gel
	Punctal plug	Scleral contact lenses	Topical epidermal growth factor, insulin, or human growth hormone
	BCL	Prosthetic replacement of ocular surface ecosystem	Albumin eye drops
	Debridement	N/A	Matrix-regenerating agent
	Tarsorrhaphy		Amniotic membrane extract drops
	Botulinum toxin A		N/A
	Fibrin glue		
	Prophylactic topical antibiotics and steroids		
Surgical	N/A	Amniotic membrane graft or transplant	
		Phototherapeutic keratectomy	

Small defects of less than 1 mm will self-heal within a day. Defects larger than 3 mm that create a loose edge can usually be placed back like a jigsaw puzzle [12]. Additionally, the placement of a bandage contact lens (BCL) serves as a therapeutic measure to minimize trauma and promote healing [1,21,39]. The use of preservative-free eyedrops and eye gel can also aid in the healing process and reduce discomfort. Most defects heal without complication. However, individuals with larger defects of >4 mm are at an increased risk of developing astigmatism secondary to epithelial hyperplasia as a response to the injury. Although large defects have a higher likelihood of causing postoperative complications, most studies show excellent long-term visual prognosis [12,40]. In Dastgheib et al.’s study, all patients had 20/30 best-corrected visual acuity (BCVA) at 12-month follow-up [41]. Similarly, in the study by Smirennaia et al., all eyes with defects larger than 4 mm attained the postoperative goal after phototherapeutic keratectomy (PTK), with patients reaching the postoperative goal at the 12-month mark [12].

Recurrent corneal erosions

LASIK

Corneal erosions are characterized by epithelial loosening that may predispose individuals to epithelial defects. In cases of recurrent epithelial erosion syndrome, the epithelium fails to adhere firmly to the underlying stroma and remains chronically loose in a specific area of the cornea, even after multiple instances of abrasion and healing.

The symptoms of corneal erosions can include sudden-onset sharp eye pain, the sensation of the eyes sticking to the eyelid, and visual complaints [40]. These symptoms may manifest acutely several months after corneal refractive surgery, and an examination may reveal either abrasions or loose epithelium. If a patient experienced an epithelial defect during the intraoperative period, recurrent erosion often occurs in the same location.

RCE is an extremely rare condition, with a reported incidence range of 0%-0.14%. Many large-scale studies do not report a single case of RCE, while other studies involving a significant number of eyes report only isolated cases [12,21,42]. Two large studies of 1,873 and 1,650 eyes report one case of RCE each, and a study of 5,566 eyes reports a 0.14% incidence [11,38,43]. Although RCE is rare in the context of LASIK, LASIK itself is a common cause of RCE. In a study of 100 eyes with RCE, 7.3% of patients developed the condition after undergoing LASIK [44]. Trauma appears to be a significant risk factor for RCE development as 39.3% of patients in the study developed RCE after minor corneal injuries. The authors proposed that abnormal healing after trauma may explain the development of RCE after minor abrasions and corneal surgery. This theory may also account for the lower incidence of RCE in femtosecond LASIK (FS-LASIK), as it avoids the risks associated with microkeratome-related epithelial defects. A study by Kremer reported an incidence of 0.04% for RCE in FS-LASIK [45]. However, RCE can still occur in FS-LASIK due to the corneal epithelial trauma caused by laser applanation and flap manipulation [46]. Additionally, LASIK procedures may exacerbate underlying RCE. Surgeons have reported incidences of epithelial fragility intraoperatively that later present with signs of RCE [45,47]. Oruçoğlu et al. report that 87.5% of patients who developed RCE had intraoperative erosion [43]. Additionally, many recurrent erosions develop in the same location as the intraoperative defect. In a case series by Ti and Tan, five out of eight eyes developed recurrent defects in the same area as corneal erosion [48]. Similar results were reported in another series of four eyes [43]. Patients with epithelial basement membrane dystrophy (EBMD) are also at much higher risk for developing RCE. In a case series by Rezende et al. of 35 eyes and 18 patients with EBMD, 27.8% developed RCE after LASIK [49]. If untreated, patients may develop other complications, such as DLK or EI [50].

PRK

Recurrent corneal erosions may be paradoxically associated with PRK and PTK, as these procedures are often performed as corrective measures for this type of complication. It is theorized that an abnormal postoperative healing process involving the deposition of extracellular matrix mediated by transforming growth factor-beta (TGF-β) and the subsequent activation of myofibroblasts is the underlying mechanism behind this complication [51].

The estimated incidence range of RCE associated with PRK is reported to be between 0.02% and 17.1%. A retrospective study by Diez-Feijóo et al. examined the incidence of various causes of RCE in 100 patients, and it was found that 17.1% of patients with RCE had a history of PRK, with the distribution of the defect being mostly diffuse. This subset of patients was also found to be younger compared to other groups experiencing RCE that was due to causes such as EBMD. The authors proposed that the method of laser ablation, mechanical scraping, or alcohol debridement during PRK could be contributing factors to abnormal healing of the stromal bed [44].

A study by Hovanesian et al. tracked symptoms in PRK and LASIK patients who were at least six months postoperative. Symptoms of RCEs were defined as eye soreness upon touch, sharp eye pain, and eyelid sticking. It was found that there was a significantly greater incidence of these symptoms in PRK patients compared to LASIK patient, particularly upon waking [42]. A case report by Busin and Meller found “dot-like changes” or microcysts in the central cornea persisting up to two years post-PRK, suggesting abnormal epithelial basement membrane healing [52]. These microcysts could potentially predispose patients to corneal erosion due to instability in the epithelial basement membrane.

There are few reported incidences of RCE in post-PRK patients [53]. In a three-year follow up of 240 eyes, Maguen et al. report four eyes that exhibited signs of RCE. Only two patients presented with both symptoms and examination findings of RCE, while another eye had only the examination findings, and one eye had the symptoms without corresponding examination findings [54]. Another study of 13 eyes after PRK noted two eyes with abnormal examination findings of elongated epithelial cells, but it was unclear if this is a sign of RCE [55]. Overall, there are limited studies reporting epithelial-related complications after PRK, which may suggest that PRK-related epithelial complications are rare [56].

The initial treatment for RCEs is conservative (Table 4). Lubricating drops and gels help reduce the likelihood of reinjury, and hypertonic saline drops help the loose epithelium adhere to the underlying stroma and promote healing [48,51]. BCLs and oral analgesics can be used for patient comfort [43]. Autologous serum drops and umbilical cord blood drops have shown superiority to artificial tears, and they are an option for patients unresponsive to initial treatment. Additionally, some patients may benefit from amniotic membranes. Topical steroids and oral antibiotics can be effective in RCE patients with meibomian gland dysfunction (MGD) by downregulating matrix metalloproteinase and lipase, which contribute to epithelial basement membrane dysfunction [51].

**Table 4:** Summary of Studies Reporting the Incidence of Epithelial Defect Associated With SMILE SMILE, small incision lenticule extraction; NP, not provided; RCEs, recurrent corneal erosions

Study	Instrument	Eyes (patients)	Percentage of the incidence of RCEs (eyes)
Hovanesian et al. (2001) [42]	Summit SVS Apex	1,731 (231)	Eye soreness to touch: 43%
			Sharp pains: 20.4%
			Eyelid sticking: 14.7%
Diez-Feijóo et al. (2014) [44]	NP	117 (100)	17.1% (20) RCEs
Maguen et al. (1994) [54]	NP	240	0.02% (four) RCEs
Mohammadpour et al. (2017) [56]	Technolas 217-Z	260 (130)	4.6% (six) RCE

If conservative treatment fails, the surgical management of RCE is the next step. Anterior stromal puncture (ASP) is recommended for peripheral RCE, although it can cause scarring and should be avoided near the line of sight [57]. Yttrium aluminum garnet (YAG) laser stromal puncture is a less extensively studied method that has shown some success and potentially has less stromal scarring. In cases where RCE involves the central cornea, epithelial debridement with diamond bore polishing (DBP) may be effective. DBP can remove disorganized and dysfunctional basement membrane, allowing for the regrowth of normal basement membrane, though patients undergoing DBP commonly require topical steroids to reduce the incidence of corneal haze. In a study by Miller et al., 47 of 49 eyes achieved symptom-free outcomes at 25.2-month mean follow-up with DBP [51]. PTK, which smooths the corneal surface and allows for specified depth of ablation, can also be performed in patients with RCE who require refractive correction [43]. The complication rates with surgical intervention are generally low, although a case of DLK occurred after diamond burr [46].

Diffuse lamellar keratitis secondary to epitheliopathy

LASIK

Several potential triggers for DLK have been identified through case reports and series, with epithelial defects being a significant risk factor associated with its development [58-66]. The average incidence of DLK associated with LASIK procedures, as calculated across multiple studies, was found to be 0.9%, with an incidence range of 0.6%-1.2% [67,68]. A study by Mulhern et al. specifically reported an incidence of DLK associated with epithelial disturbance at 0.6%. After retreatment, all cases achieved an UDVA of 20/20 or better, with no recurrence of DLK [69].

In another study of 1,000 eyes by Hoffman et al., 1.1% of eyes developed corneal abrasion with subsequent DLK [61]. After a mean postoperative follow-up of 5.2 months and treatment with steroids, there was no loss of BCVA, and 10% of eyes gained one or more lines of BCVA. Additionally, Shah et al. found that patients with epithelial defects experienced a 24-fold increase in relative risk for the development of DLK [70]. The higher incidence of DLK in patients with epithelial defects may be attributed to inflammation related to wound healing. Mirshahi et al. found that 91% of patients with severe epithelial defects developed DLK, while the overall incidence of epithelial defect associated DLK was 1.2%. Moreover, 21.1% of patients achieved preoperative BCVA scores 4-8 months after initial surgery while 15.8% lost two or more lines of Snellen visual acuity [11].

Similarly, chronic epithelial loosening and recurrent abrasions can prolong ocular surface inflammation, thus explaining the higher DLK incidence in patients with RCE. In one case series by Haw and Manche, all eyes that developed RCE also presented with DLK. Corneal refractive surgery can predispose patients to DLK following subsequent trauma, even years later. In LASIK, there have been reported cases of DLK occurring in patients who developed corneal abrasions with an average postoperative period of nine years [71]. DLK incidence is also increased in patients with basement membrane abnormalities. In a study involving patients with EBMD by Pérez-Santonja et al., 54.5% of eyes developed subsequent DLK [23]. Considering that DLK can be a consequence of epithelial-related defects such as abrasions and erosions, supportive treatments including the use of artificial tears, antibiotics, and additional measures such as BCLs remain important in managing DLK.

SMILE

DLK secondary to epithelial defect in SMILE appears to be rare. Reinstein et al. reported that 0.10% of 4,000 consecutive eyes that underwent SMILE had epithelial defect-related DLK. This study is currently the only available source reporting DLK secondary to epitheliopathy in SMILE, and the reported incidence was considered as the average. Postoperative UDVA was 20/20 or better, and there was no loss of any lines of BCVA (distance) or change in contrast sensitivity [72]. The treatment approach for DLK in SMILE is identical to that for DLK following LASIK procedures.

Epithelial ingrowth

LASIK

EI is a rare complication of LASIK characterized by epithelial invasion into the flap-stromal interface. During flap creation, a corneal epithelial-stromal pathway is introduced to the ocular surface. During post-LASIK healing, proliferating epithelial cells may migrate into the flap interface, leading to ingrowth. Ingrowth may be self-limiting and asymptomatic in most cases; however, ingrowth can sometimes obscure vision or cause astigmatic changes. Astigmatic changes are more likely to occur after flap melt, a complication of EI. EI can also be associated with flap melt, which is the keratolysis of flap tissue secondary to collagenase released by hypoxic, intra-flap epithelial cells [72].

The reported incidence range of EI in primary LASIK is from 0% to 3.9% with lower incidence observed for FS-LASIK compared to m-LASIK [73]. A comparative study of LASIK with zero-compression Hansatome to femtosecond flap creation reported an EI incidence of 0.2% and 0.1%, respectively [21]. Another study by Kohnen et al. focused solely on FS-LASIK, involving 1,210 eyes, reported an EI incidence of 1.6% [1]. The lower incidence of EI in FS-LASIK may be attributed to reduced trauma during flap creation. It is theorized that epithelial trauma near the flap edge contributes to abnormal epithelial proliferation and subsequent ingrowth [74]. Flap lift enhancement surgeries have a higher incidence of postoperative EI (10%-20%) [73]. Techniques aimed at minimizing trauma may reduce the incidence of EI. A small study by Chan and Boxer Wachler where surgeons delineated the flap edge prior to flap lift showed a lower EI incidence [74]. This procedure likely reduces epithelial trauma compared to directly lifting the flap with forceps, which can cause epithelial tearing at the flap edge. FS-LASIK also appears to have an advantage in flap lift enhancements, as evidenced by Letko et al. who reported less EI in FS-LASIK cohort compared to mechanical LASIK cohort [75].

Epithelial defects are associated with increased incidence of EI. One study of 3,786 eyes that underwent LASIK by Wang and Maloney documented an incidence of 0.92% (35 eyes) of EI, with 14 of which showing EI associated with epithelial defects [76]. Flap edge trauma eases epithelial access to the flap-stromal interface. Additionally, epithelial defects trigger a more robust healing response, amplifying epithelial proliferation [47]. A study by Pérez-Santonja et al. reported that 72.7% of eyes with large epithelial defects (>20% of flap surface) developed EI, and 36.4% had subsequent flap melt [23]. Another study of 16 eyes post-LASIK reported an EI incidence of 50% with flap melt occurring in four eyes with corrective scraping of the interface epithelium [40]. Other risk factors that may affect wound healing such as diabetes are associated with increased incidence of EI [48].

The treatment of EI depends on the severity of visual compromise (Table 4). The Probst classification is commonly used to grade EI. Grade 1 is characterized by thin and difficult-to-detect growth and typically does not require treatment. Grade 2 involves epithelial nests affecting the flap edge, while grade 3 exhibits pronounced growth with haze around the flap edge. Grade 4 EI is characterized by cells invading the visual axis along with flap melt. Grades 3 and 4 require urgent treatment [45]. Mechanical debridement, with or without adjunctive agents, is the main modality for treating EI. Adjunctive therapy includes ethanol (dilute: 50%, 70%, and 100%), 0.02% mitomycin C (MMC), flap suturing, fibrin glue, and amniotic membrane. The recurrence of EI can occur in 0%-36% of patients, so it is important to inform patients about the possibility of retreatment. The evidence suggests that debridement with fibrin glue has the lowest rate of recurrence (0%-7.9%) [48].

The prognosis for EI varies depending on the severity of vision compromise. Grade 1 EI that does not affect vision may only require observation. For grade 2-3 EI, 77%-91% of patients achieve 20/40 BCVA after treatment. For grade 4 EI, flap amputation and penetrating keratoplasty may be necessary to improve visual outcomes [48].

SMILE

EI is also rare in reported literature for SMILE with estimated incidence range of 0.016%-0.5%. EI can occur postoperatively when epithelial cells invade through the lenticule incision or intraoperatively through the seeding of epithelial cells during suction. The progression of these ingrowths can lead to visual disturbances, reduction in visual acuity, and foreign body sensation. The risk factors for EI may include cap rupture, diabetes mellitus, tears of the incision, or epithelial dystrophy of basement membrane. EI incidence in reported literature varies. Moshirfar et al. reported an incidence of 0.5% post-SMILE in one study [21]. Another study by Wang and Maloney of 6,373 eyes reported one case of interface ingrowth that was debrided at seven months postoperative and recovered [76]. Kamiya et al. report two cases of EI treated with debridement without flap lifting because it occurred near the incision. By one month, the best spectacle-corrected visual acuity (BSCVA) for these patients improved to 20/16 or more without complication [77]. Thulasi et al. report one case of recurrent EI after difficult lenticule extraction during SMILE. The patient was not responsive to debridement and suture, so hydrogel ocular sealant was attempted with no recurrence. The patient had undiagnosed diabetes mellitus type 2, which may have contributed to epithelial dysfunction and delayed healing [78]. Micro-distortions have been observed in the corneal epithelium including Bowman’s layer via OCT post-SMILE, but no significant long-term visual regression was found [79,80]. One study by Shetty et al. found that intraoperative cap repositioning could reduce the risk of these distortions [81].

The treatment of ingrowths includes irrigation and removal via vitreoretinal forceps or spatula with topical antibiotics and steroids thereafter. One documented case utilized hydrogel ocular sealant after the scraping of the ingrowth [78]. This sealant may be prophylactic against ingrowth. A case report by Nijdam et al. suggested that the application of MMC, ethanol, fibrin glue, proparacaine, BCL, or low-energy neodymium-doped yttrium aluminum garnet (Nd-YAG) laser after the removal of the ingrowth could be effective [82]. A novel method for the treatment of advanced central epithelial ingrowth has been successfully conducted by Kankariya et al. [83]. The method utilizes CIRCLE (Carl Zeiss Meditec AG, Jena, Germany) software to create a “cap to flap” conversion, allowing greater and safer access to the original interface site so that the ingrowth can be excised. Thus, there is less corneal epithelial damage due to less difficulty in gaining entry to the corneal insult.

Microstriae

LASIK

Striae, or folds in the flap tissue, can occur when the flap is unable to lie completely flat against the underlying stroma. Microstriae, observable on confocal microscopy, are technically present in all patients. However, visually significant striae are much less common, with an estimated incidence ranging from 0.033% to 4.6% [1,84]. Various factors can contribute to the development of striae, including irregularities in the ablation surface, edema, flap dislocation, debris, and epithelial defects [85]. Striae can induce corneal irregularities and cause a range of symptoms, including loss of visual acuity, glare, foreign body sensation, and astigmatism. The risk factors for striae include higher ablation depth, age greater than 50, endothelial cell dysfunction, epithelial erosions, and EBMD [86].

Epithelial defects significantly increase the incidence of striae. In a study by Kohnen et al., 55% of patients with severe epithelial defect developed microfolds [1]. There may be an underlying mechanism that predisposes certain populations to microfolds. In a study of over 100,000 surgeries, Wallerstein et al. reported that 23.5% of eyes with clinically significant striae occurred bilaterally [84]. Furthermore, the incidence of striae in patients with known underlying EBMD is extremely high compared to the population average (18.2%) [42].

SMILE

In a study conducted by Wang and Maloney, which examined 6,373 eyes to assess postoperative complications after SMILE, a reported incidence of 0.14% for microstriae was observed, with most cases being identified intraoperatively. Notably, none of these cases had any effect on corrected distance visual acuity (CDVA) [76].

There are several management options available for flap striae. In the largest study on post-LASIK striae by Wallerstein et al., the authors reported lifting the flap at the slit lamp, removing remnant epithelium on the stroma, and irrigating to remove striae [84]. This technique yielded an 80% success rate at immediately resolving striae with further improvements over time. The surgeons of a different study opted to treat striae at the operating table with a sterile technique. The flap is similarly lifted and irrigated, but the inner surface of the flap was also debrided if large striae were noted. This technique was able to help 74% of patients achieve target visual acuity at 12-month follow-up [86]. Other techniques have been described to treat striae including massaging and smoothing with a cotton swab, BCL, refloating, or PTK [85]. If there are persistent striae, astigmatism may develop. PTK has been effective in treating microstriae with persistent visual symptoms. In a small study of 47 eyes, 81% of eyes achieved 20/33 or better BCVA, and the results remained stable at 24-month follow-up [87].

Filamentary keratitis

PRK

Corneal refractive surgery can also be a risk factor for filamentary keratitis (FK). The average incidence of FK in the literature is reported to be 5.4%, with an incidence range of 1.5%-3.8%. Theoretically, corneal excision from surgery can undergo abnormal healing with eventual epithelial detachments from the basement membrane. These areas of detachment act as a nidus for epithelial cells and mucus leading to the formation of filamentous structures that can cause pain and foreign body sensation.

FK has been associated with PRK. For example, a study by Mohammadpour et al. investigated the incidence of FK in post-PRK patients wearing a BCL for different durations. They found that patients who wore the BCL for seven days had a lower incidence of FK (3.1%) compared to those who wore it for four days (7.6%). The logarithm of the minimum angle of resolution (LogMAR) UDVA and CDVA were significantly lower in the group with seven days of BCL versus four days of BCL (P=0.016 and 0.001, respectively); however, the UDVA and CDVA were not significantly different after three months (P>0.05). The authors theorized that greater BCL duration prevented eyelid friction that could induce more epithelial detachment and FK progression [56].

Treatment options for FK include filament removal using artificial tears, topical steroids, or topical diclofenac sodium. Other treatment modalities may include the use of N-acetylcysteine, BCLs, punctal occlusion with plugs, botulinum toxin injections, or rebamipide. These options can be employed based on the severity and individual patient characteristics [88,89].

Discussion

Corneal refractive surgery is subject to both intraoperative and postoperative epithelial-related complications. These complications include epithelial defects, RCEs, microstriae, EI, DLK, and FK. These complications arise due to epithelial tissue insult and aberrant healing processes.

The incidence of epithelial-related complications can vary depending on the type of refractive surgery. SMILE, which does not involve flap creation or mechanical scraping, has been reported to have a lower average incidence of epithelial defects (3.3%) compared to LASIK (4.9%). Mechanical LASIK, which uses a microkeratome, has a higher average incidence of corneal defects (4.7%) compared to femtosecond LASIK (FS-LASIK) with an average incidence of 1.9%. (Figure 4) [13,21]. The risk factors include advanced age, fair skin, blue eyes, topical anesthetic, and thicker cornea, with the most contributory risk factor being prior history of intraoperative epithelial defect during LASIK [10-13,28]. Many of these complications are associated with intraoperative defects. For example, corneal abrasions or erosions are a major risk factor for DLK, corneal striae, and epithelial ingrowths [11,60,90]. The modification of surgical techniques to reduce corneal damage and controlling risk factors that impair corneal healing such as diabetes are of utmost importance in reducing these complications [4].

PRK, despite being a treatment for recurrent corneal erosions, can also predispose patients to RCEs, with an estimated incidence ranging from 0.02% to 17.1%. The specific technique used during PRK, such as corneal scraping, laser ablation, or alcohol application, may influence epithelial healing and the occurrence of erosions [41]. More studies are needed to determine how techniques can influence the risk of RCE as LASIK-related RCEs are rare, with an incidence range of 0% to 0.14%.

Overall, the visual prognosis for epithelial-related complications is favorable with appropriate treatment for epithelial defects, RCEs, DLK, FK, and EI. Postoperative improvements in uncorrected visual acuity (UCVA) and BCVA for each of these epithelial-related complications were present in the literature. Nonetheless, EI appeared to have less favorable outcomes with higher-grade EIs in 77%-91% of patients achieving a BCVA of 20/40 with grade 2 or 3 EI post treatment. Additionally, the rate of EI occurrence can be as high as 36% [73].

A limitation of this study is that despite comprehensive literature search, incidence ranges and the average incidence of epithelial defects across all categories (erosions, abrasions, EI, DLK, FK, etc.) and all surgeries (PRK, LASIK, and SMILE) could not be calculated or obtained. Perhaps this could be due to the mechanism of each type of corneal refractive surgery or surgical experience, thus leading to various studies with differing rates of epithelial-related complications across different surgeries.

Treatment options for epithelial-related complications vary and can include both surgical and medical management (Table 4). Mild epithelial defects may only require supportive care with lubricating eye drops or hydrating contact lenses. However, severe defects may require corrective procedures such as PRK, PTK, or surgical excision.

## Conclusions

Given the frequency of corneal refractive surgeries, it is essential for clinicians to assess the risk of both intraoperative and postoperative epithelial-related complications. Surgical techniques, medical treatments, and patient-specific risk factors should all be considered to optimize patient safety and postoperative outcomes. Ongoing research is focused on investigating the efficacy and safety of various treatment options for these complications.
